# Nitrous Oxide

**Published:** 1872-08

**Authors:** James B. Hodgkin

**Affiliations:** Alexandria, Virginia.


AMERICAN JOURNAL

OF

DENTAL SCIENCE.

Vol. VI. THIRD SERIES—AUGUST, 1872. No. 4.

### ARTICLE I.

### *Nitrous Oxide.*

BY JAMES B. HODGKIN, D. D. S., ALEXANDRIA, VIRGINIA.

Review of Article in New York Medical Journal, Aug., 1870, on Nitrous Oxide as an Anæsthetic, by R. Armory, M. D. of Mass.

[CONCLUDED FROM PAGE 350, VOL. IV.]

Our author certainly deserves credit for the careful manner in which he has conducted his experiments, for the accuracy of his observations, whether it shall be seen in the end that he has established his proposition, that "anæsthesia by Nitrous Oxide is due to capillary stasis," or not, no one will refuse him the thanks of the profession for his investigations into a subject over which so much acknowledged mystery hangs.

According to some observers Nitrous Oxide acts in an analagous manner to Carbonic Oxide. It does not exalt vitality. Sleep, stupor, coma, are not evidences of exalted vitality, but the reverse, as is apparent to any reflecting mind. Although normal sleep, indeed results in renewed vitality, yet its own condition is manifestly lower in vital force than wakefulness. A simple illustration will suffice here: we all know how much more susceptible to malarious influences a sleeping person is than one who is awake, a susceptibility manifestly due to lowered vitality as present during sleep.

Carbonic Oxide acts on the blood by devitalising the red blood corpuscles. It seizes the oxygen present in them and appropriates it, and not only so, but presents more oxygen from being taken up by the blood. This condition may tend to produce the "capillary stasis" of our author.

There is probably going on in the blood during anæsthesia a variety of changes, and a number of conditions may be present, intangible and undetectable; it is not improbable that anæsthesia may at some time be ascertained to consist of not one condition, but of several, their ultimate result being capillary stasis, and other abnormal conditions. Anæsthetics may act directly on the muscular tissue of the small arteries, diminishing their calibre; thus lessening the amount of blood supplied to the brain. This is the theory of the action of Bromide of Potash.

An accumulation of effete matters in the blood will produce stupor and if anæsthetics prevent the elimination of these they will add to this stupor. Whenever the process of the oxydation of the blood is arrested or retarded there is an accumulation of these foreign matters in the system, and this may readily aid in the process of anæsthesia. We all know how promptly used and other substances eliminated by the kidneys, retained in the system by disease of these organs, will produce coma, and further how chloroform and ether administered internally have a sedative effect. So that it seems probable that anæsthesia is the result of various agencies, and not of one alone.

How Nitrous Oxide produces capillary stasis, if it does do so, we may never know, but it seems probable that it is due to more causes than one. That stasis is always present I am inclined to doubt, for numbness of the extremities is present in only a part of the cases falling under my observation. It is difficult to see how it can be present in those cases where struggling and screaming were incidental to the operations for which the gas was administered. With complete or nearly complete stasis, it is difficult to see how this could result.

The statement often made that Nitrous Oxide acts in a contrary manner to chloroform,—acts as an excitant, is worthy of a moment's notice. Certainly the pulse is excited in the one and depressed in the other case, but if it is remembered that the vapor of the chloroform displaces from the lungs scarcely an appreciable quantity of air, while Nitrous Oxide entirely displaces the contained air and completely fills the lungs, I think the reason for the exaltation will be seen. Filling the lungs with a compound unnatural to them stimulates an eager desire for air, respiration is hurried and large, and the heat is in turn excited to increased actions; but there seems to be no certainty that stimulation other than this takes place. True that excitation takes place when a few whiffs of gas are taken, and air intermixed, but this proves nothing, for opium taken in small doses excites, and in large doses is sedative.

